# Crohn’s disease-related anal fistula cancer diagnosed by examination under anesthesia: a case report

**DOI:** 10.1186/s40792-023-01722-8

**Published:** 2023-08-23

**Authors:** Daisuke Kaneshiro, Yuusuke Sanechika, Kazuki Kishi, Daichi Sakai, Kazuya Iwamoto, Mitsunobu Takeda, Yujiro Nakahara, Tomofumi Ohashi, Atsushi Naito, Kenta Furukawa, Jeongho Moon, Mitsunobu Imasato, Tadafumi Asaoka, Tsunekazu Mizushima

**Affiliations:** Department of Gastroenterological Surgery, Osaka Police Hospital, 10-31, Kitayamacho, Osaka-Shi Tennoji-Ku, Osaka, 543-0051 Japan

**Keywords:** Anal fistula cancer, Crohn’s disease, Inflammatory bowel disease, Colorectal cancer, Anorectal cancer, Examination under anesthesia

## Abstract

**Background:**

As the number of patients with inflammatory bowel disease (IBD) increases, the incidence of IBD-related colorectal cancer (CRC) is also on the rise. Crohn’s disease (CD)-related CRC has been reported to have a poorer prognosis than sporadic CRC, and the early detection of CD-related CRC is difficult. Japanese patients with CD are reported to have a higher frequency of anorectal cancer than the Western population; however, methods for early diagnosis have not yet been established because of perianal pain during the examination.

**Case presentation:**

We report a case of CD-related anal fistula cancer that was detected early by surveillance examination under anesthesia (EUA). The patient was a 37-year-old man, diagnosed with CD at the age of 15 years and started medical treatment. However, due to poor disease control, the intestinal tract remained highly inflamed and the patient continued to have over 10 bowel movements per day. He was referred to our hospital for surgical treatment after a colonoscopy (CS), which revealed multiple active ulcers and stenoses. Since three perianal seton drainage tubes had been placed around his anus since the age of 33 years, we decided to perform an EUA to rule out cancer coexistence in the anorectal region. After a random biopsy of the rectum by CS under general anesthesia, we resected and curetted multiple perianal fistulas as much as possible and reinserted the seton drainage tubes. Pathological examination of the fistula tract revealed adenocarcinoma in one tract, indicating the coexistence of anal fistula cancer. Based on the diagnosis of multiple intestinal stenoses and anal fistula cancer due to CD, we performed hand-assisted laparoscopic total colectomy, rectal amputation, extensive perineal resection, and reconstruction using a left rectus abdominis flap.

**Conclusion:**

In a long-term CD patient with anorectal lesions, we performed an EUA to diagnose the coexistence of anal fistula cancer at an early stage, and surgical resection was achieved. EUA is effective for the early detection and treatment of CD-related CRC and may contribute to an improved prognosis.

## Background

The number of patients with Crohn’s disease (CD) has increased in recent years. With advances in medical treatment, the prognosis is relatively good, with a cumulative survival rate of 96.9% at 10 years after diagnosis [1].

However, digestive cancer complications are the main cause of the excess mortality associated with CD. In CD patients, the standardized mortality ratio for all causes and CD-specific causes is high, especially for small intestine and colorectal cancers (CRCs) [2].

The 5-year survival rates for CD complicated by malignancy have been reported as follows: Stage I (88%), Stage II (68%), Stage IIIa (71%), Stage IIIb (25%), and Stage IV (0%) [3].

CD complicated by advanced cancer has a poor prognosis, and early detection is critical. However, because of intractable anal lesions and intestinal stenosis, imaging evaluation is tough, making early detection difficult. Colonoscopy (CS) is often difficult to perform because of stenosis, and capsule endoscopy is not commonly performed.

In this report, we describe a case of CD-related anal fistula cancer that was diagnosed early through surveillance, and surgical resection was performed.

## Case presentation

The patient was a 37-year-old man. He was diagnosed with CD at the age of 14 years. He was started on infliximab at age 26 years, followed by azathioprine at age 31 years, and budesonide at age 33 years, but his disease remained poorly controlled.

At the age of 35 years, he underwent seton drainage for his anal lesions and subsequently switched from infliximab to adalimumab, but the inflammation of the intestinal tract remained completely uncontrolled. At the age of 37 years, he was referred to our department for the surgical treatment of multiple stenoses of the intestinal tract and anorectal area.

He had no medical history, was 164.9 cm tall, weighed 47.8 kg, and had a body mass index of 17.6.

Physical examination revealed no tenderness in the abdomen, but he had 10 bowel movements per day, and 3 seton drainage tubes were placed around the anus. Outpatient rectal examination could not be performed due to pain. Blood test results showed anemia (Hb: 8.6 mg/dL) and low nutrition (albumin: 2.5 g/dL, prealbumin: 15 mg/dL); inflammatory markers were mildly elevated (CRP: 3.54 mg/dL).

**Fig. 1:**
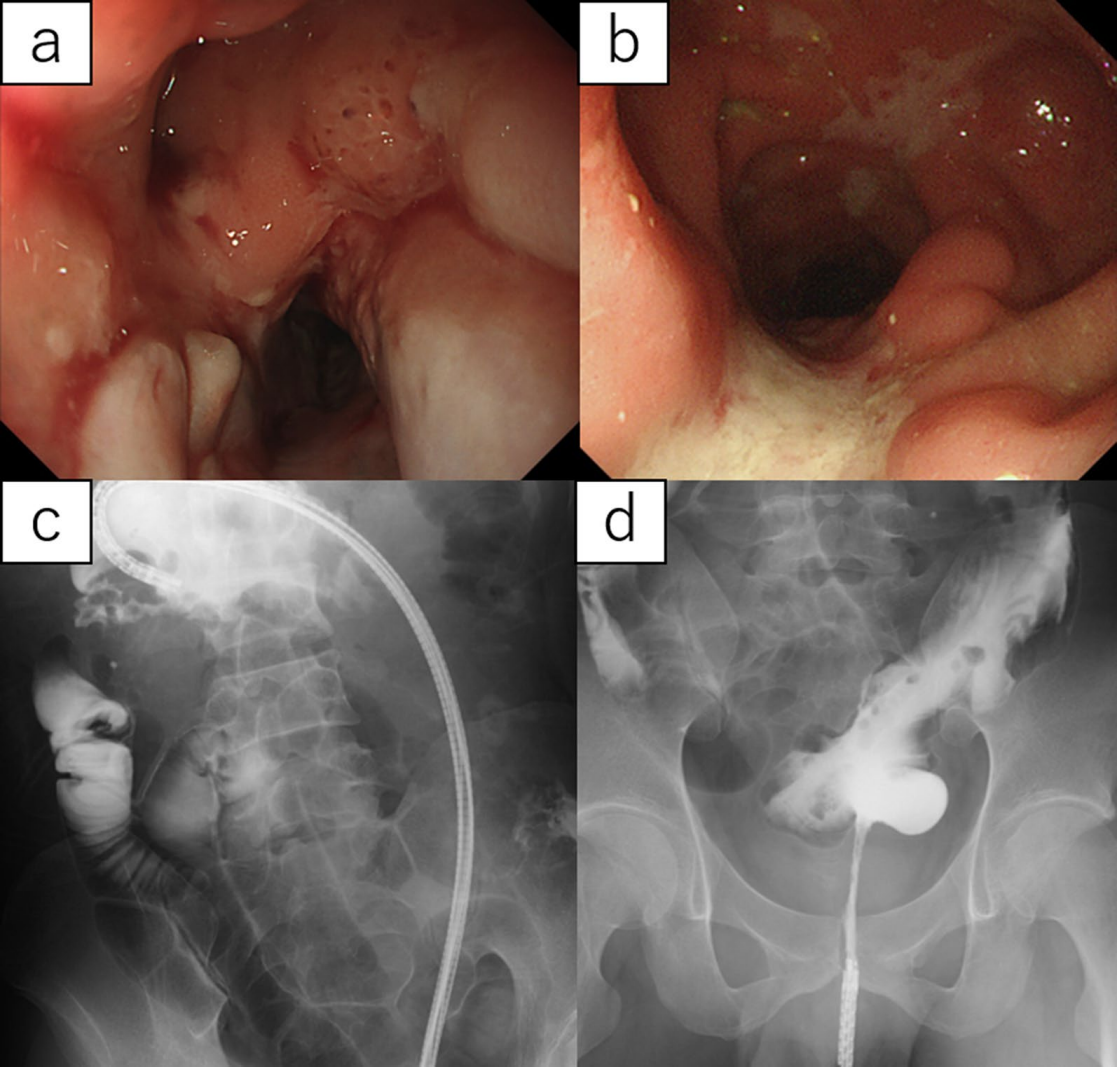
Preoperative findings of the CS and endoscopic contrast examination. CS shows a cobblestone appearance and longitudinal ulcer (**a**, **b**), and endoscopic contrast examination reveals severe stenosis in the transverse colon (**c**) and rectum (**d**)

CS showed a cobblestone appearance, a longitudinal ulcer, and multiple stenoses that were difficult to pass without a transnasal endoscope (Fig. 1a, b). Endoscopic contrast examination revealed severe stenosis in the transverse colon and rectum (Fig. 1c, d), which was considered an indication for surgery. The Montreal classification was A1L3B2p, and the CDAI was 283.

**Fig. 2:**
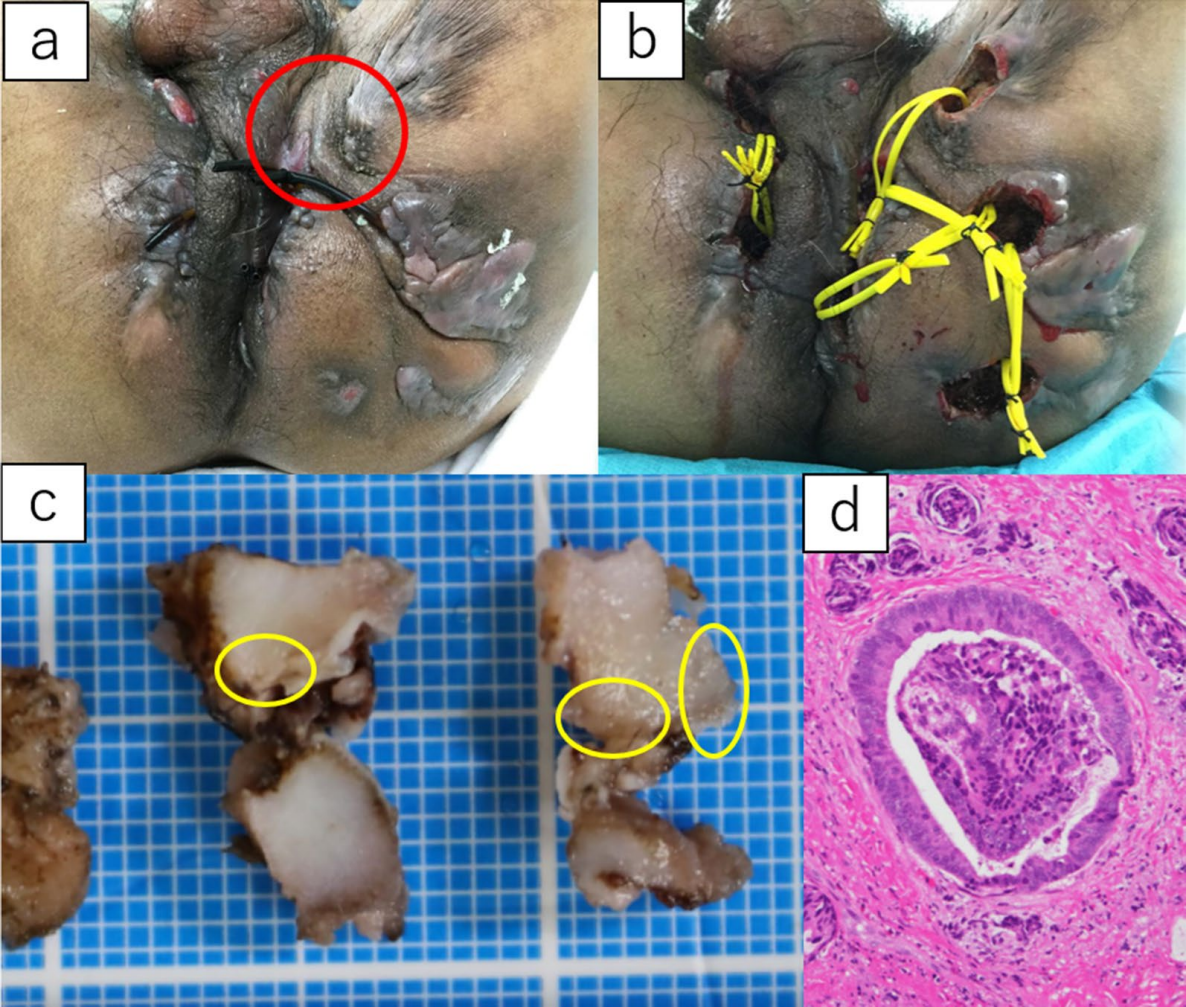
Preoperative and postoperative condition of the perianal area and the findings of the pathological examination. **a** Before EUA; **b** after EUA; **c** picture of the specimen (anal fistula cancer was detected from the area encircled); **d** microscopic findings of the specimen

Prior to surgery, we decided to perform an examination under anesthesia (EUA) to exclude perianal malignancy. Rectal examination under general anesthesia reveals the anal fistula has one primary lesion and three secondary ones. One seton drainage tube was placed between the primary and secondary, and two seton drainage tubes were placed between the secondary lesions (Fig. 2a). Following this, endoscopy was performed, and a random biopsy was obtained from the stenosis of the rectum, which was difficult to evaluate in detail during the preoperative examination. The perianal infected foci were scraped, and the fistula tract was resected as far as possible and subjected to pathological examination. The operation was terminated by the reinsertion of a seton drainage tube (Fig. 2b).

Pathological examination revealed no malignancy in the random biopsy of the rectum, but part of the resected fistula revealed a highly differentiated adenocarcinoma, and the patient was proven to have coexisting anal fistula cancer (Fig. 2c, d).

Contrast-enhanced CT and contrast-enhanced magnetic resonance imaging studies were performed, but the perianal area showed severe inflammatory changes, and the spread of the lesion could not be evaluated. No obvious lymph node or distant metastasis was observed. Tumor markers (CEA: < 1.7 ng/mL, CA19-9 < 2 U/mL) were within the normal range.

Finally, we diagnosed the patient with CD-related anal fistula cancer (cT1N0M0 cStage I) and performed hand-assisted laparoscopic total colectomy, rectal amputation, extensive perineal resection, and reconstruction with a left rectus abdominis flap.

**Fig. 3:**
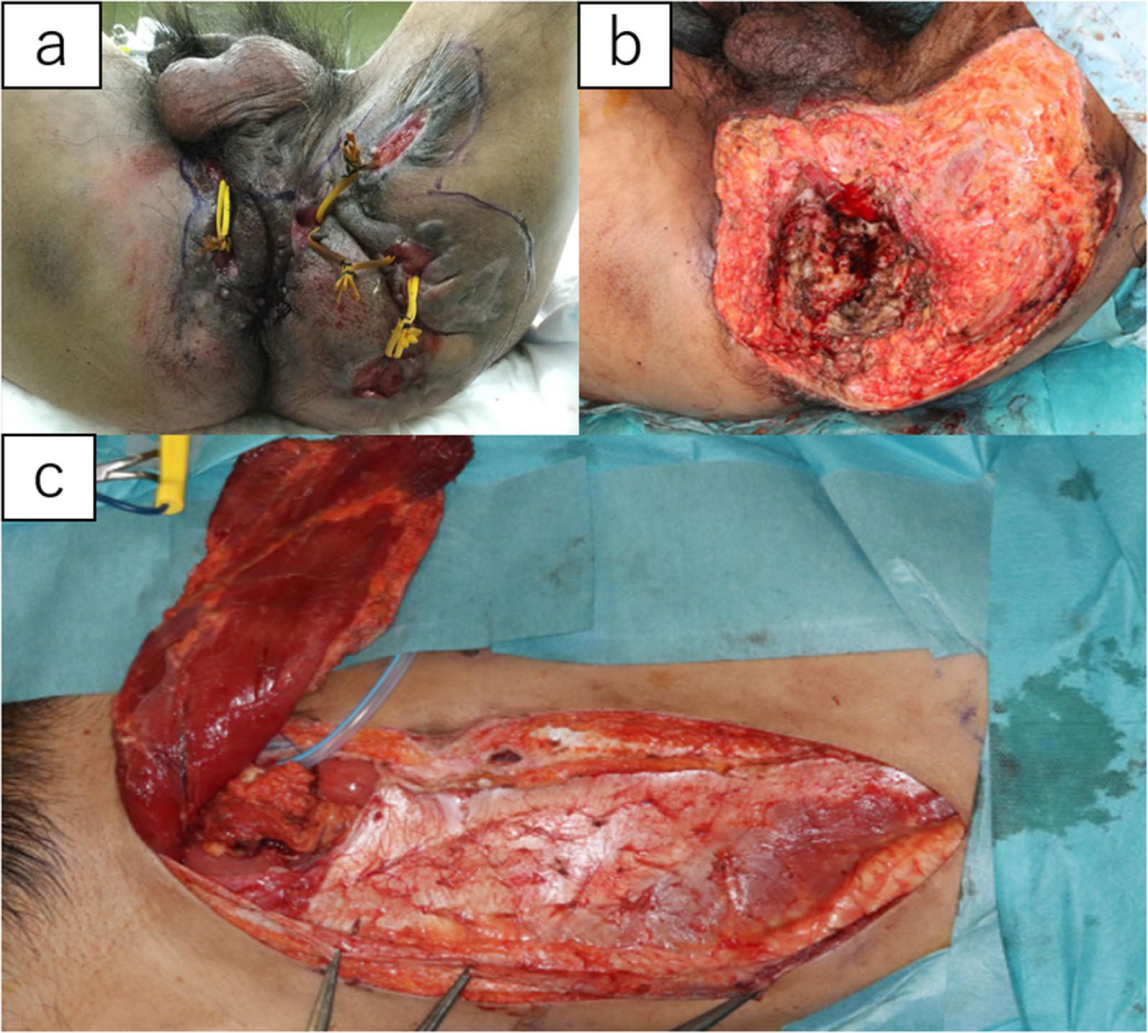
Operative findings. **a** Preoperative marking; **b** after APR and extensive perineal resection; **c** left rectus abdominis flap

**Fig. 4:**
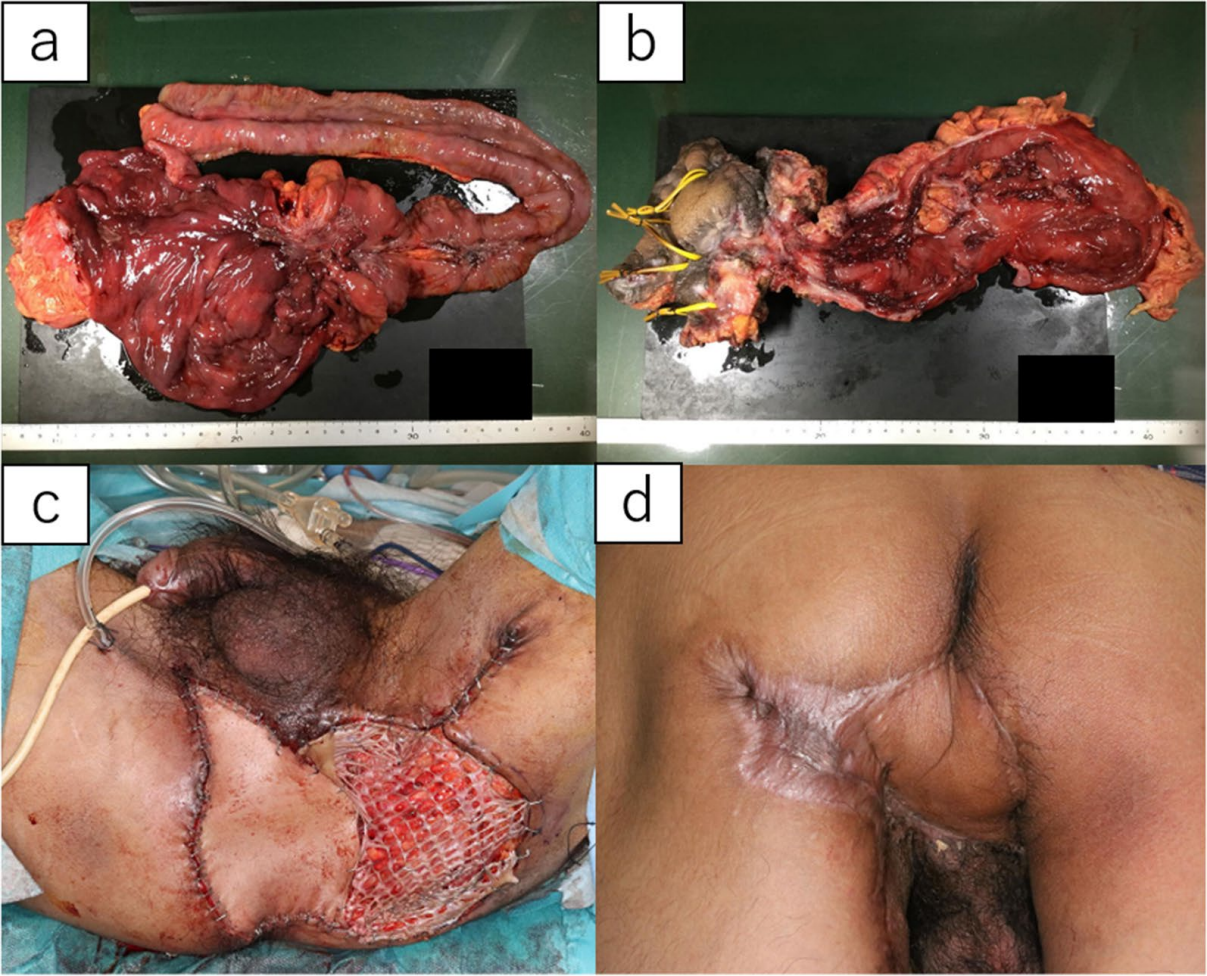
Specimen and postoperative course of the perianal skin defect. **a** Right hemicolon; **b** left hemicolon; **c** just after the operation; **d** at postoperative day 150, most of the wound area was epithelialized

After total colectomy and partial rectal resection with hand-assisted laparoscopy, the patient underwent a transanal procedure. We performed a resection of the remaining rectum and an extensive perineal resection to remove the fistula as much as possible (Fig. 3a, b). The perineal tissue defect was replaced with a left rectus abdominis flap (Fig. 3c), and the remaining skin defect on the left buttock was treated with a segmental skin graft (Fig. 4c). The operative time was 12 h and 5 min, and the blood loss was 1280 mL. A urethral injury was found on postoperative day 14, but the patient recovered conservatively with the insertion of the urethral catheter. On postoperative day 44, the patient was discharged home.

Although the skin graft fell off, the skin flap survived, and epithelialization was achieved in most of the wound area 3 months postoperatively (Fig. 4d).

Pathological examination showed that the anal fistula cancer extended 4 cm in length, with a histology of well- or moderately differentiated tubular adenocarcinoma and mucinous adenocarcinoma. The pathological diagnosis was pT2N0M0 pStageIIA (UICC-TNM classification, 8th edition).

 10 months postoperatively, the patient has had no apparent recurrence.

## Discussion

In Europe and the United States, meta-analyses have shown that patients with CD with widespread bowel inflammation are at high risk for developing CRC as well as ulcerative colitis (UC) [4, 5], and CD-related CRCs are reported to have poorer prognosis than UC-related CRCs [6]. While right-sided colon cancer is reported to be more common in Europe and the United States [7, 8], anorectal cancer, including anal fistula, is more common in Japan. CD-related cancers in Japan occur most frequently in the anorectal region, including anal fistula cancer, which accounts for 51% of all cases, followed by rectal cancer (29%) [3]. However, perianal examination of CD patients is often difficult because of pain and bowel stricture, and EUA with rectal examination and biopsy of the lesion has been proposed as a surveillance method.

CD-related colorectal cancer in Japan is characterized by (a) cancer onset at a young age (around 50 years old), (b) a long disease duration of more than 10 years, (c) invasive types (types 3, 4, or 5), (d) a tendency for poorly differentiated types (mucous carcinoma, poorly differentiated carcinoma), and (e) a predilection for the anorectal area [9]. Based on these characteristics, it has been proposed that patients with the disease for at least 10 years undergo surveillance examinations every 12–24 months. In cases where cancer complications are suspected, repeated examinations over a shorter period may be considered.

Only nine cases of surgical resection of CD-related anorectal cancer have been reported in the English literature (Table 1) [10–18]. In addition, six cases of surgical resection of CD-related anorectal cancer diagnosed by EUA have been reported in the Japanese literature. In all of these cases, the disease duration was > 10 years, and abdominoperineal resection (APR) and total pelvic exenteration (TPE) were performed in most cases. Cancer surveillance revealed that the total detection rate of CRC was 6.19%, and the detection rate was 1.85% with core needle biopsy, 5.56% with endoscopic biopsy, and 5.88% with excisional biopsy [19].

**Table 1:** Reports of surgical resection of CD-related anorectal cancer

Year	Author	Age	Sex	Years of CD	Location of cancer	Histological type	Trigger of diagnosis	Diagnostic method	pTNM (pStage)	Operation	Prognosis (months)
2005	Devroe et al.	70	Female	25	Anal fistula	–	Examination of symptoms	–	–	APR	–
2008	Tokunaga et al.	57	Female	11	Anal fistula	tub2	Growth of perianal tumor	Biopsy	–	APR	> 24
2012	Bautista et al.	41	Male	–	Anal canal	muc	Examination of symptoms	Biopsy	–	–	–
2013	Sogawa et al.	40	Male	19	Anal canal	tub1	Examination of symptoms	Biopsy with CS	pT2N0M0	APR	> 42
2014	Scharl et al.	48	Female	25	Anal fistula	muc	Growth of perianal tumor	Biopsy	ypT2N0M0	APR	–
2016	Maejima et al.	50	Female	33	Anal canal	muc	Examination of symptoms	Biopsy	pT2N0M0	APR	> 18
2018	Pareja-López et al.	42	Male	20	Anal canal	Hydroadenocarcinoma	Examination of symptoms	Biopsy	–	Tumor resection	–
2020	Ehrl et al.	54	Male	20	Anal canal	Verrucous carcinoma	Large perianal tumor	Biopsy	–	APR	–
2022	Komono et al.	47	Female	27	Anal fistula	muc	Examination of symptoms	Biopsy	–	TPE	–
2022	Our case	37	Male	23	Anal fistula	tub1 > tub2 > por	Screening	Biopsy	pT2N0M0	APR	> 9

At our institution, patients with CD who have had the disease for more than 10 years are actively screened for malignancy using EUA. We performed EUA in the lithotomy position under general anesthesia and CS after visual inspection and rectal examination. Because of rectal and anal stenosis, CS is frequently difficult, and we collaborate with and frequently perform procedures with endoscopists. In addition to the evaluation of stricture and biopsy of ulcerative lesions and fistulas, we performed random biopsies in the rectum and anal canal, even in the absence of obvious lesions. Finally, we resected the fistula as much as possible and submitted it to pathological examination to rule out the complication of anal fistula cancer. The surgery was concluded with the placement of seton drainage.

EUA is expected to be useful for the early detection and curative treatment of difficult-to-diagnose CD-related anorectal cancers. However, EUA has not been used in a long time, and there is a scarcity of data. Thus, it is necessary to examine the efficiency, usefulness, and medical economy of EUA by including more cases in the future.

## Conclusions

Herein, we report a case of CD-related anal fistula cancer that was diagnosed early by the EUA.

## Data Availability

All data generated or analyzed during this study are included in this published article.

## Abbreviations

CS: Colonoscopy
CRC: Colorectal cancer
CD: Crohn’s disease
EUA: Examination under anesthesia
IBD: Inflammatory bowel disease
UC: Ulcerative colitis
APR: Abdominoperineal resection
TPE: Total pelvic exenteration
